# The value of credit in science

**DOI:** 10.1007/s13194-026-00731-2

**Published:** 2026-03-20

**Authors:** Thijs Ringelberg

**Affiliations:** Faculty of Philosophy, Department of Theoretical Philosophy, University of Groningen, Groningen, the Netherlands

**Keywords:** Credit economy, Credit incentives, Scientific motivation, Esteem, Praise, Values in science

## Abstract

The Credit Economy Approach (CEA) explains scientific behaviour in terms of credit-seeking: scientists pursue social credit, which furthers their careers. This paper investigates the relationship between such credit incentives and the normative motivations often attributed to scientists, such as epistemic and ethical commitments. A default assumption – which I call the *cumulative view* – holds that these are distinct sources of motivation, each exerting independent influence. Drawing on a conceptual analysis of social credit and findings from social psychology, I argue instead for an *entangled view*: credit incentives operate through, and depend upon, shared normative commitments. On this view, the credit economy functions in virtue of a scientific community’s shared evaluative norms. This account deepens existing work that situates the credit economy within a broader social context, and offers a framework for further research integrating formal work in the CEA, conceptual analysis in philosophy of science, and empirical insights from the social sciences.

## Introduction

Why are you reading this paper? Perhaps you’re engaged in a pursuit of truth. Alternatively, you might hope to improve your standing in your academic community. More likely, it’s some combination of the two.

This paper investigates the relationship between credit incentives and the normative motivations often attributed to scientists, such as epistemic and ethical commitments. A default assumption – which I will call the *cumulative view* – is that these are distinct sources of motivation, each exerting independent influence. I argue instead for an *entangled view*: credit incentives operate through, and depend upon, shared normative commitments. Drawing on social psychology, I show that the credit economy functions only in virtue of evaluative norms that are internal to scientific communities.

The Credit Economy Approach (CEA) is a prominent approach in formal social epistemology of science. Over the past thirty-five years, it has grown from initial formulations by David Hull and Philip Kitcher (Hull, 1988; Kitcher, 1990, 1993) into a full-fledged formal modelling tradition where theorists engage in debates over the social functioning of science on the basis of analyses of mathematical models of scientific communities.

In these models, scientists are represented as seekers of *social credit* (Bright, 2021). Credit is distributed on the basis of the priority rule: the first person to make a finding, gets all the credit for it (Strevens, 2003). Credit has instrumental value: it can be used to ‘buy’ career-furtherance (Strevens, 2013). The resulting notion of a *credit economy* has proven explanatorily powerful. It has aided our understanding of a range of scientific phenomena – for example communism, which is the tendency to share results amongst scientists (Heesen, 2017); pluralism, the tendency of scientists to take various approaches to a given problem (Kitcher, 1990; Strevens, 2003); scientific misbehaviours such as plagiarism and fraud (Bright, 2021; Heesen, 2024); and systemic disadvantage faced by historically underrepresented groups in science (Rubin & Schneider, 2021).

Despite the explanatory power of the CEA, few theorists would maintain that scientific motivation can be exhaustively understood in terms of credit incentives. Such an incentives-only reading is only defended by Boettke and O’Donnell (2016), but only in the normative sphere: they argue that the only responsibility of economists is to pursue career advancement, and that the scientific institutions should be organised accordingly.

Most theorists stress that scientists are motivated by more than just credit-incentives. Scientific motivation has normative aspects, too: epistemic values, like the desire to find truth, and more ethical values, like the belief that scientists ought to share their work. Hugh Desmond (2021), for example, argues that credit-incentives cannot by themselves adequately explain the default trust that can be observed amongst scientists, indicating that a full explanation of the scientific process must involve normative or ethical considerations alongside considerations of credit incentives. On the more epistemic side, Kevin Zollman (2018) argues that scientific rationality demands that scientists have “mixed motivations”: they should not merely hunger for credit, but also aim to achieve truth.

Arguments like these raise a question: how do credit-incentives relate to the normative aspects of scientific motivation?

A default answer is the cumulative view, according to which credit incentives and normative aspects are distinct domains that have separate effects on the scientific process. On this view, the effects of credit incentives can be studied in isolation from the effects of the normative aspects of scientific motivation. The arguments of Desmond and Zollman might be read as exhibiting an implicit commitment to this cumulative view. For example, Zollman, before landing on the superiority of a mixed view, considers both an “only-credit” and an “only-truth” view of scientific motivation – thereby apparently implying that he thinks it possible to neatly separate credit incentives from epistemic aspects. Desmond similarly distinguishes between ethical or normative considerations on the one hand, and credit on the other.

Recent work by Rubin and Schneider (2021) challenges the assumption that credit incentives can be isolated from broader social factors. They argue that network dynamics in a scientific community, which are influenced by the community’s social history, can affect the way credit is doled out. A credit economy studied in isolation from its social history, then, will behave differently from a credit economy studied in tandem with it.

In this paper, I argue a similar point for the normative aspects of scientific motivation: against the cumulative view, I argue that credit incentives are entangled with these normative aspects, which are social in nature. This means that credit incentives studied in isolation from the scientific community’s epistemic and ethical values will behave differently than credit incentives studied in tandem with those values. Just like in Rubin & Schneider’s argument, this entanglement is partially mediated by social-psychological effects. Credit-seeking, I argue, should not be understood as a selfish activity in which scientists engage alongside ethical and epistemic activities: when we investigate the nature of credit-seeking, we find that it is inextricably linked with the ethical and epistemic aspects of science. The credit economy functions in virtue of a shared set of norms and values that are endorsed by the members of a scientific community.

This entanglement thesis goes beyond the idea, promoted by authors like Kitcher (1990) and Strevens (2003), that credit incentives are aligned with the epistemic ends of science. This alignment thesis argues that credit incentives cause behaviour that serve the normative ends of science, but it says nothing about the conditions under which those incentives take shape. My entanglement thesis focuses on those very conditions: it argues that the credit economy is an inherently social structure, consisting of a shared set of norms and values that underpin and give rise to the incentives of the credit economy.

The arguments I offer in this paper have a number of applications. First, while the CEA stresses the incentive-based aspects of scientific behaviours, I emphasise that these incentives are rooted in shared norms and values. This suggests that these norms and values should be taken into account when formulating incentive-based interventions of the kind the CEA tends to promote: how far can we stretch the incentive structure before it starts hampering the normative basis on which it rests? Second, I argue that our understanding of the relevant norms and values could be informed by insights from social psychology, which will allow us to further strengthen the empirical basis of this branch of the social epistemology of science – thereby going some way toward addressing criticisms that the CEA and similar modelling approaches lack empirical validation (Cartwright, 2005; Martini & Fernández Pinto, 2017; Thicke, 2020).

I begin, in Sect. 2, with a conceptual analysis of the notion of credit. I argue that to better understand this notion, and thus to better understand the functioning of the credit economy, we require a refined account of the prestige-related processes of science – which I provide in Sect. 3. There, I argue that the credit economy is a representation of an intricate social structure, an interplay between scientific institutions and evaluative social judgements that scientists make about each other, and that this interplay depends on the existence of a normative consensus about what it means to be a good scientist. In Sect. 4, I further investigate this normative consensus. I argue that the consensus must be substantive: it refers to values that are analytically distinct from career-incentives. I also show that social-psychological social identity theory offers us the theoretical tools to understand the nature of that consensus. In Sect. 5, I discuss an important objection: why do I not ground the CEA in well-known sociological analyses of the prestige-related processes of science? I conclude by sketching how the account I present can serve as a framework for future inquiry into the social organisation of science.

## What is social credit?

Upon reading CEA-accounts, one quickly gets an intuitive grasp of the notion of credit. Anybody who operates within academia is familiar with the idea that they need to perform in order to advance (e.g. Strevens, 2013, 22). But only very rarely is this intuitive grasp interrogated further: what exactly *is* credit? This is the question which this section and the next aim to answer.

The main purpose of the present Sect. 2 is to get an overview of the aspects of credit that require explaining. I start, in subsect. 2.1, by offering two premises that jointly summarise the role of credit in the CEA. In subsect. 2.2, I scrutinise the various aspects of the world that theorists have pointed to in order to describe credit. And in subsect. 2.3, I consider the role our own intuitions play in our understanding of credit. I conclude that in order to understand the nature of credit, we require a theoretical account of the prestige-related processes of science – the construction of which will be the project of Sect. 3.

### Two premises of the credit economy approach

The role of credit in the CEA can be characterised by means of two premises:

***Premise 1: Credit-career link***

*Social credit is the means by which scientists advance their careers.* 1

***Premise 2: Intertemporal regularity***

*Credit is awarded predictably for the same kinds of behaviours/achievements across time.*

Assuming that all scientists have an interest in moving up in academia (or at least in remaining a scientist), premise 1 entails that all scientists have an interest in maximising^2^ the credit they receive.

The regularity referred to in premise 2 is usually taken to be described by the priority rule: the first scientist to make a contribution gets all the credit for it.^3^ Sometimes, however, theorists experiment with other “reward systems” (e.g. Strevens, 2003; Zollman, 2018). To be able to count such approaches as instances of the CEA, I leave the regularity unspecified in my formulation of premise 2.

### Characterisations of credit

While the premises tell us how credit behaves, we can still ask: what *is* credit? What is this *thing* which is awarded predictably, and which can be used to further a scientist’s career? Table 1 provides an overview of the terms used by various theorists to describe the notion of social credit and its predecessors.

From one perspective, the great diversity of this list is striking. Clearly esteem, citations, knighthood, and the other items in Table 1 aren’t the same thing. But from another perspective, we can clearly see that they are related: all point to the social world of science. Specifically, they all refer to science’s processes of *social status*, which we might call science’s prestige-related processes.

**Table 1:** Various descriptions of credit

Theorist	Operative term(s)	Specifications
Robert Merton	Recognition	Eponymy; prizes; representation in the iconography of medals; honorary academies; peerage; knighthood; mention by historians. (Merton, 1957)
Bruno Latour & Steve Woolgar	Credit, credibility	“(…) scientists’ abilities actually to do science”. (Latour and Woolgar, 1987, 198)
David Hull	Credit, status, recognition, reputation (Hull, 1988, 281)	Referenced use of one’s ideas. (Hull, 1988, 283)
Paula E. Stephan	Recognition (Stephan, 1996, 1201)	See Merton
Michael Strevens	Prestige	“reputation, a sizable office, the rapt attention of graduate students, and the like”. (Strevens, 2003, 57)
Liam Kofi Bright	Social Credit	Honour; esteem; glory; status; being found impressive; publication in acclaimed journals. (Bright, 2021)
Remco Heesen	Social Credit	Citations; (job) appointments; prizes. (Heesen, 2019)

So here is a proposal: the notion of credit is a way to cleanly represent these prestige-related processes – however they may function – by abstracting away from their intricacies. This is not an uncommon method: for example, in game-theoretic analyses of hunting strategies the pay-offs might be cast in terms of “nutritional value”, abstracting away from the various processes of food intake and the various foods involved in these processes.^4^ If we take a similar view of credit, then the fact that the notion covers so many things is a strength, not a weakness.

### Intuitions about credit: the plagiarism prize

Even so, the terms in Table 1 are too broad: not every instance of a prize, citation, or eponym counts as credit. Some such “rewards” are detrimental for one’s career. Think of eponyms like the Wakefield affair (Maisonneuve & Floret, 2012), Hwanggate (Saunders & Savulescu, 2008), or Lysenkoism; citations in retraction notices or expressions of concern; or prizes like the Rusty Razor Award, awarded to the “worst promoters of pseudoscience”, who sometimes are practicing scientists (Marshall, 2021, 2023). Presumably, none of these count as social credit in the sense intended by CEA-theorists.

And we can readily produce more examples. Heesen suggests that we can increase the amount of credit scientists receive for high-impact work by awarding prizes for it – thereby incentivising scientists to produce such work (Heesen, 2019, 9). This seems to be an application of a more general principle: awarding a prize for X-behaviour increases the credit gained with X-behaviour – prizes are a form of credit, after all.^5^ But would awarding a plagiarism prize incentivise plagiarism? Probably not: a plagiarism prize seems to fall in the same category as the Rusty Razor Award, in the sense that receiving it is quite undesirable.

How do we tell citations, eponyms, and prizes that can be represented as credit from those that can’t? The knowledge is rather intuitive: as an academic, I simply know that I would rather not receive a retraction notice or a Rusty Razor. I have such intuitions because I am intimately familiar with the social world of science and its prestige-related processes, being part of the academic community myself. To further our understanding of credit, it would be helpful to make these intuitions explicit.

What we need, then, is a theoretical account of the prestige-related processes of science that can replace our intuitions. This account will make explicit the inherently social nature of the credit economy by specifying the social mechanisms that are involved in its functioning. The account will set conditions on the way we use social credit in our models, just like our intuitions do, but in a transparent way – meaning that we can interrogate the mechanisms by which conditions are set. To salvage the CEA as it has been developed thus far, the account should mirror these intuitions as closely as possible, thereby allowing us to use the notion of credit similarly to how we have been using it. The plagiarism prize will be a good test case in that respect. The project of the next section is to construct this account of the prestige-related processes of science.

## The prestige-related processes of science

Constructing this account is a peculiar theoretical endeavour: we must, as it were, replace the foundations of the CEA-building whilst ensuring it remains upright. Thus while the account I sketch is about the (very real) prestige-related processes of science, empirical accuracy is not my primary goal: instead, the main aim of my account is to capture the central aspects of the CEA as outlined in the previous section. I take a functionalist approach, aiming to answer the question: how can the prestige-related processes of science function in such a way that they are aptly represented by the Credit Economy Approach? Our account of the prestige-related processes of science must therefore ground the central aspects of the CEA as outlined in the previous section: the two premises of subsect. 2.1; the various descriptions of credit in Table 1 (subsect. 2.2); and the intuitions of subsect. 2.3.

### Attitudes and institutions

To start, note that the descriptions of credit provided in Table 1 can be grouped into two kinds. Some can exist only in the context of a formally organised social institution: publications only in the context of journals; sizable offices only in the context of universities. I call these *institutional* descriptions of credit. The remainder are social phenomena that do not require formal organisations. In order to stay true to the methodological individualism that typifies the CEA (see Sect. 5), I interpret these as either social attitudes themselves (such as esteem or attention) or as grounded in those attitudes (such as prestige or reputation; see also the discussion of prestige in the next subsection). I refer to them as attitudinal descriptions of credit.

I will use this two-fold dependence of credit on attitudes and institutions as the basis of the account. The prestige-related processes are an interplay between certain attitudes of members of the scientific community, and certain institutions of that community. While the attitudes explain the two premises, the institutions explain our intuitions. I discuss the attitudes first, in subsect. 3.2; then value-measures, which are a precondition for the attitudes, in subsect. 3.3; and then the institutions in subsect. 3.4. In subsect. 3.5 I conclude by finally providing an answer, on the basis of our freshly constructed account of the prestige-related processes, to the question with which we started: what is credit?

### Attitudes: esteem and prestige

On the attitudinal side, the picture I sketch is inspired by the notion of an economy of esteem introduced by Brennan and Pettit (2005). This is the basic idea: the credit economy functions because scientists frequently evaluate each other in their capacity as scientists. To be evaluated positively is to be esteemed. Prestige is an inherently social phenomenon that emerges as a result of many members of a community esteeming the same individual. This social phenomenon, prestige, is instrumentally valuable to scientists, because it will naturally lead to improved career opportunities: to be considered a good scientist is to be a sought-after candidate. This explains the first premise, that social credit is the means by which scientists advance their careers.

Brennan and Pettit define esteem as an evaluative attitude of an individual toward another person, where that person is evaluated positively. Esteem is based on a specific evaluative measure: to esteem someone is not to deem them good in general, but to deem them a good piano player, orator, or rock climber. It is merely an attitude: esteem does not require actions such as praise or approving looks – although it will often lead to them (Brennan & Pettit, 2005, 16–23).^6^

This notion of esteem captures an evaluative element which can be recognised in all the attitudinal entries in Table 2. We find someone *impressive* to the extent that we have a positive evaluative attitude toward them. Latour and Woolgar, who introduce the notion of *credibility*, say that credibility “concerns scientists’ abilities actually to do science” (Latour and Woolgar 1987, 198): to find someone credible is to consider them good (or at least capable) scientists. *Recognition* is similar: we recognise our peers when we consider them exemplary of our community in some way. Even the concept of *credit* itself is evaluative, for it is the opposite of blame: while we blame people for their bad deeds, we credit them for their good ones.

**Table 2:** Attitudinal and institutional characterisations of credit

Attitudinal	Institutional
Prestige; reputation; the rapt attention of graduate students; credibility; recognition; honour; esteem; glory; status; being found impressive	Eponymy; prizes; medals; honorary academies; peerage or knighthood; mention by historians; (referenced) use of one’s ideas; a sizable office; having one’s work published in an acclaimed journal; having one’s work cited; job appointments

A challenge seems to be posed by *the rapt attention of graduate students*: attention is not intrinsically evaluative. But mere attention cannot function as credit: a professor who sets her hair aflame during a lecture will capture the rapt attention of her graduate students, but it does not make her a prestigious scientist, and her actions will not earn her increased career opportunities. The attention needs to be of the right kind, and the right kind of attention is based on positive evaluative attitudes.

For the remaining attitudinal descriptions – *honour*, *status*, *reputation*, *glory*, and *prestige* – mere esteem is insufficient. The scientist esteemed by a single peer is not prestigious, nor does she bask in glory. *Recognition* and *credit* can fall into this same category if they are used in a more general sense, for example in the sentences “X is recognised for XYZ” or “X is credited with XYZ”. Other than esteem, the notions in this category are not privately held mental attitudes, but rather inherently *social* phenomena that can only emerge in groups. A key condition for their emergence is coordination of esteem: things like honour, status, and general recognition emerge when a sufficiently large portion of the community esteem the same individual. Following Rubin and Schneider (2021), who similarly conceptualise of prestige as an accumulation of individual judgements, I will refer to this as “prestige”.

### Value-measures

The existence of prestige in a scientific community requires that esteem-attitudes of significant portions of the scientific community coincide: there must be some *inter-esteemer regularity* in the allocation of esteem. How is this possible? The answer to this question will help us explain premise two, that credit is awarded predictably for the same kinds of behaviours/achievements across time.

An evaluative attitude requires a value-measure: only if I can tell good from bad behaviour can I esteem or disesteem my peers (Brennan & Pettit, 2005, 103).^7^ If attitudes of esteem of a number of people coincide, as prestige requires, then a reasonable conclusion is that the value-measures underlying their esteem coincide too. Of course, I have not yet considered the contents of the value-measure underlying scientific esteem: I will do so in Sect. 4. As a placeholder, we might say that the value-measure for scientific esteem is some notion of the good scientist. All we know about this notion at present is that the attitudinal side of the prestige-related processes of science, in order to function in such a way as to be aptly represented by the CEA, requires a degree of consensus in the scientific community about it – whatever its contents may be. I will therefore also refer to this notion as a community’s *normative consensus*.

This normative consensus explains premise two, intertemporal regularity in the allocation of esteem. The distribution of esteem and prestige is regulated by a shared concept of a good scientist. Conceptual change is slow: the notion of a good scientist will not suddenly change from one day to the next. It therefore makes sense to expect intertemporal regularity in the allocation of esteem and prestige: it will be awarded consistently for similar achievements across time.

Note that this means that credit is community-relative: what counts as credit for an individual depends on which value-measure she endorses, which in turn depends on which normative consensus she participates in. This issue parallels what Carole Lee (2020) calls the reference class problem for credit valuation in science. Consider, in this context, also the discussion below (subsect. 4.3) of community-relative notions of the good scientist.

### Institutions: praise

An esteem-based account of the attitudinal side of the prestige-related processes explains the attitudinal descriptions of credit and both premises of the CEA. This leaves the institutional descriptions of credit and our intuitions about what counts as credit-incentivisation. Both are addressed in this section.

I argue that the role of institutions in the prestige-related processes of science is to mitigate informational problems. Esteem-judgements require evidence about esteem-relevant behaviours: in order to tell whether you’re a good pianist, I need to hear you play. But in the large community of science, it is impossible to get all such evidence first-hand. Institutions solve this problem by transmitting information about scientists’ esteem-relevant behaviours across the community.

Certain scientific institutions are perceived by members of the community as providing evidence of esteem-relevant behaviours of scientists, as stipulated by the normative consensus. Think of prize committees, journals, and the citation system. I call these “praise-institutions”. The information these institutions provide – prizes, publications, citations, and the other institutional entries in Table 2 – is called “praise”. Since praise can be easily transmitted, it allows esteem and prestige to spread through the community more easily: merely from seeing that a certain scientist has garnered many citations, for example, her peers may conclude that she is a good scientist – but only on the condition that they see citation numbers as evidence of a scientist’s goodness. And due to the career-improving nature of prestige, praise can lead to improved career opportunities.

This notion of praise aligns with received ideas about credit-institutions. Heesen and Bright (2021) distinguish short-run credit (such as publication) from long-run credit (such as citations or prizes). Both, they argue, represent an “estimate of the real epistemic value of a contribution to science” (p. 646), but long-run credit does a better job at this, because it is both a more considered and a more democratic opinion. Their analysis supports the idea that credit is based on individual epistemic evaluations filtered through institutional mechanisms.

The notion of praise-institutions allows the account to explain our intuitions about plagiarism prizes. A high-impact prize transmits information about something which scientists (presumably) deem good behaviour: winning the prize constitutes a message that this scientist has delivered high-impact work. It is a praise-institution, and can therefore be represented as credit. A plagiarism prize, however, transmits information about something which scientists (presumably) deem bad behaviour: winning it is a message that a scientist has misbehaved. It isn’t a praise-institution. A plagiarism prize does not lead to prestige, nor to improved career opportunities. A plagiarism-prize thus does not constitute an incentive to commit plagiarism, and cannot be represented in CEA-models models by means of credit.

### So what is credit?

We have constructed an account of the prestige-related processes that explains the central aspects of the CEA. This means that if the prestige-related processes function in the way the account specifies, then the CEA is an apt representation of them. The account paints the prestige-related processes as having two aspects: esteem and praise. But which of these aspects can be called credit?

The answer is: both of them. The interactions between esteem and praise conjure up an image of a cycle of value making its way through a scientific community in various forms – now esteem, now praise – to be eventually cashed in in terms of career opportunities. One motivation for scientists to engage in scientific activities might therefore be the expectation of improved career opportunities in the future. The CEA is a representation of this idea, representing both esteem and praise by means of a single concept: credit. This allows it to abstract away from the intricacies of the prestige-related processes.^8^

But the notion of credit does not cover all aspects of esteem and praise. Due to its strategy of abstraction, the CEA can represent the incentive-aspect of scientific action with exceeding clarity. But this clarity comes at a cost: the CEA abstracts away from the evaluative and normative aspects of the prestige-related processes, even though those aspects play an important role in the coming to be of those same incentives. Thus if we want to do more than merely analyse the consequences of a particular incentive structure being in place – if we wish, for example, to specify the conditions under which that incentive structure is in place; to understand the way the incentives interact with norms and values; or to assess the limitations of certain incentive-based interventions – it will be helpful to turn to the account of the prestige-related processes which we have just constructed. The last section of this paper is devoted to teasing out some of the qualities of this account. As we shall see, the account can further our conceptual understanding of the social processes of science. It also provides us insight into where empirical input is required to further our knowledge about these social processes.

## Values in the credit economy

The account of the prestige-related processes that we have constructed in the previous section exhibits an entangled view of credit incentives and normative aspects of scientific motivation: for the credit economy to function, the community must have a shared conception of the good scientist, on the basis of which esteem is won and praise (ideally) gets doled out. We have seen *where* this notion plays a role, and *how* it does this: the notion of a good scientist functions as an evaluative measure on the basis of which scientists esteem each other. But we have not yet considered the contents of the notion. Is a good scientist just interested in furthering their career, or is there more to it than that? In this section I scrutinise the nature of the ideal of the good scientist, thereby deepening the entangled view. The section also functions as a showcase: it displays the uses to which the esteem-praise account can be put by social epistemologists of science.

In subsect. 4.1, I discuss how career-incentives and the normative consensus interrelate. In subsect. 4.2, I argue that we must understand the normative consensus to be substantive: it makes reference to values which are in principle independent of the self-interest of scientists. In subsect. 4.3, I discuss what kinds of values might be contained in the normative consensus. In subsect. 4.4, lastly, I argue that we can understand the normative consensus as a social-psychological social identity. I conclude that the esteem-praise account shows that the social identity of scientists is *epistemically relevant*: the epistemic functioning of the community depends on the (contingent) shape of this social identity.

### Two faces of scientific action

The notion of the good scientist is a double-edged sword. By serving as the evaluative measure for esteem-judgements it plays an indispensable role in the credit economy, furnishing scientists with career-incentives. But it is also a norm of good behaviour for scientists. Since this norm must be widely accepted in a scientific community for the credit economy to function at all, scientists in this community can also choose to act on it directly.

This means that many scientists – all those who participate in the normative consensus – have two distinct ways of deciding what actions to engage in. The first is to follow the incentives of the credit economy: to decide based on the career prospects offered by the various options. The second is to follow their normative convictions: to act as they believe a good scientist should. The prestige-related processes of science cause the verdicts of these two decision rules to coincide – at least under idealised conditions.^9^

The career-incentives are spawned by the normative consensus: it is because scientists can expect most selection committees to select candidates on the basis of approximately the same criteria (namely, those stipulated in the normative consensus) that there are strong enough links between careers and scientific activities to base one’s choice of activities on those links. The normative conception of scientific action is therefore analytically prior to the incentive-driven one. This means that the social processes of science would keep functioning adequately if all scientists only followed their normative convictions, and nobody let themselves be guided by career-incentives. Science also keeps functioning if some scientists become *alienated:* an alienated scientist does not participate in the normative consensus, but lets her actions be guided merely by career-incentives.^10^ But it is not possible for all scientists in a community to be alienated: if the normative consensus disappears from the community, then so do the career-incentives.^11^

The idea that the normative conception of scientific action is analytically prior to the incentive-driven one will be surprising to a reader familiar with Kitcher’s *The Division of Cognitive Labor* (1990). Kitcher argued that a community of “ruthless egoists” – scientists who pursue credit – outperforms a community of epistemic angels who merely pursue truth. This seems to directly contradict my statement that the social processes of science would keep functioning adequately if all scientists only followed their normative convictions. But note that this tension only arises if we assume that the normative convictions in question are the pursuit of truth. It is a good thing, therefore, that the contents of these convictions remain to be specified: so far, we have merely given them the placeholder name of a notion of the good scientist (see subsect. 3.4). I will argue in subsect. 4.3 that we should fill this notion in with a different value, which I will call *discovery*.

### A substantive conception of the good scientist

Let’s turn to the question at hand, then: what does the normative consensus about the good scientist look like? The simplest option is to stipulate that the good scientist ought to pursue the furtherance of her career. This is a norm: the stipulation that a scientist ought to pursue career-furtherance can serve as the basis of criticisms and commendations of any scientist.^12^ But the normativity is *merely formal*: content-wise, it is simply the universalisation of the private intention to pursue one’s own interests. Under this conception the coincidence of normatively guided and career-incentive-driven behaviour in science would not be surprising: it is merely a matter of definitions.

In opposition to this *merely formally normative* conception of the good scientist, we can pose *substantive* conceptions of the good scientist. A substantive conception is fashioned in such a way that the conception does not analytically reduce to the injunction to pursue one’s own interests: it refers to independent values. Under such a conception, the coincidence of normatively guided and incentive-driven conceptions of scientific behaviour would be more than just a definitional matter.

There is a good reason to reject the merely formally normative conception of the good scientist: it cannot adequately fulfil the functions we have stipulated for it in our account of the prestige-related processes of science. This is due to the double-edged character of the conception of a good scientist: it is a norm for good behaviour, and also the evaluative measure on the basis of which career-furtherance is allocated. A merely formally normative conception of the good scientist would therefore be self-referential: the good scientist is a scientist who pursues career-furtherance, and career-furtherance is distributed to good scientists. Under such a conception it is hard to see how scientists could be motivated in their activities by the prospect of career-furtherance, because the only certain way to further their career would be by furthering their career, for which they would need to further their career, and so on. Under a merely formally normative conception of the good scientist, the prestige-related processes of science do not give rise to action-guiding credit-incentives.

This is a significant finding. Merely formally normative conceptions of the good scientist have been seriously proposed, for example by Boettke and O’Donnell, who argue that “the only social responsibility of economists is to maximize their career advancement within the community of economists” (Boettke & O’Donnell, 2016). In the introduction, I referred to this as the incentives-only reading of the CEA. Zollman (2018) has offered arguments against the incentives-only reading from a normative perspective: a community of scientists solely motivated by credit performs less well than a community of scientists with mixed motivations. The argument I offer goes one step further: I argue that it is *incoherent* to think that an entire scientific community could be guided merely by a norm which stipulates that scientists ought to advance their careers, because under such a norm, the notion of career-furtherance remains undefined.

Since explaining the functioning of the credit economy is the main goal of this paper, we must thus adopt a substantive conception of the good scientist: a conception which refers to values that are analytically distinct from career-furtherance. But this raises a new question: what kinds of values are contained in the conception of the good scientist?

### What is a good scientist?

A traditional conception of a value-driven scientist is the scientist as an epistemic agent. Such an agent pursues scientific values such as truth or knowledge. This idea can be found in Kitcher, when he talks about scientists as pursuing the “high-minded goals of individual rationality” (Kitcher, 1990, 14). John Elster calls it “the pure scientist”, saying that “the formulation of a true belief is the ultimate goal of his behaviour." (Elster, 2016, 17) We might be tempted to identify the substantive conception of the good scientist which underlies the credit economy with the notion of the pure scientist.

But this approach won’t fly. In early CEA analyses, theorists like Kitcher and Strevens show that communities of credit-seeking scientists outperform communities of pure scientists (Kitcher, 1990; Strevens, 2003). The behaviours of pure scientists, then, diverge from those of credit-seeking scientists. Since the behaviours of the good scientist for which we are looking should coincide with the behaviours of credit-seeking scientists,^13^ the good scientist cannot be identified with the pure scientist: the good scientist cannot be someone who pursues the high-minded goals of individual rationality.

The CEA itself suggests an alternative. Credit is distributed according to the priority rule: the first to make a certain contribution to the field gets the credit. The more important the contribution, the more credit is won. Runners-up receive no credit at all. Given our account, we can reinterpret this: scientists tend to esteem those amongst them who make important contributions to their fields. This suggests a substantive value of the kind we are looking for: a good scientist makes important contributions to her field. We might call this value “discovery”. Under discovery, runners-up are not esteemed because they did not *contribute* anything: the finding was already there.

Although it is the only value we can infer from the CEA, there is no reason to think that scientists only esteem each other on the basis of discovery. Other values are likely to be contained in the notion of a good scientist. The notion of a good scientist might also differ between fields. Introspection into the reasons why we esteem our fellow academics provides some possible examples of other values. As a philosopher, I am impressed by those who ask interesting questions at conferences – or those who answer uninteresting questions in an interesting way. A biologist might be impressed by the ability to combine field knowledge about organisms with the ability to turn that knowledge into testable theories. Which exact values are part of a scientific (sub-)community’s conception of the good scientist is an empirical question, and the answers might vary between communities.

### An epistemically relevant social identity

We have established a range of properties of the conception of the good scientist which drives the prestige-related processes of science. The conception serves a dual function: it can guide action, but it can also be the basis of esteem-judgements. It is substantive: it makes reference to independent values. Its exact shape is specific to a scientific (sub-)community. Besides being *pre*scriptive, it can also be taken in a *de*scriptive sense: the fact that scientists value and possess (some of) the qualities stipulated in the normative consensus is what makes them scientists. The normative consensus tells us something about who is a member of a scientific subcommunity.

In all these characteristics, the normative consensus about the good scientist fulfils the function of what is called a *social identity* in social psychological self-categorisation theory. Social identities are thick concepts: they both describe the members of a certain social group, and prescribe proper behaviour in case one is acting as a member of that group (Spears, 2011; Turner, 1991). A scientist is someone who makes discoveries (descriptive aspect), and a good scientist makes discoveries (prescriptive aspect).

The scientific social identity could have been different. Scientists might have valued hard work over discovery, or they might have thought explaining scientific theory to the general public more important. The exact shape the scientific social identity has taken is a historical accident. Simultaneously, the functioning of the credit economy crucially depends on this contingent shape. If scientists were not impressed by important discoveries, then there would be no priority rule. Since the priority rule plays an essential role in CEA-explanations of such epistemic virtues as communism and pluralism (Heesen, 2017; Kitcher, 1990; Strevens, 2003), this provides us with reason to think that these virtues would be less ubiquitous in the absence of the value of discovery.

This means that the social identity underlying a scientific community is *epistemically relevant*: the contingent shape of the social identity affects the community’s epistemic performance. In some respects, the identity may be *geared toward the common epistemic good*: the fact that scientists value discovery, as we’ve seen, contributes to the ubiquity of pluralism and communism, helping the community to efficiently produce knowledge. In other respects, the identity may be *turned away from the common epistemic good*, for example if there is a belief that a good scientist is a man, causing a failure of the community to capitalise on the epistemic potential of women.

To be clear, this does not mean that scientists value the things they value *because* of their effects on the common good. A scientist who finds discovery impressive might find that her justification comes to an end there: discovery is impressive because, well, it simply *is*. She will esteem her colleague for a discovery not because he has contributed to the common good, but *because discovery is impressive*. Still, the identity in which her judgement is grounded is geared toward the common epistemic good in this particular sense.

## Sociological accounts of credit

In this paper, I have constructed a theoretical account of the prestige-related processes of science. Some readers might object, however, that I have been reinventing the wheel. The centrality of the notion of credit in modern social epistemology of science is partially an inheritance from sociological studies of science in the second half of the twentieth century. In light of these accounts, the thesis of entanglement of incentives and norms that I defend in this paper is old news. Why did I not simply use one of these sociological accounts of credit as a foundation for the CEA? My answer is that while these sociological analyses are deeply valuable, the CEA as a modelling tradition has drifted so far from its sociological predecessors that they can no longer serve as a theoretical foundation for it.

The foundational analysis of the normative structure of science derives from Robert Merton (Merton, 1973). Merton’s analysis had two aspects: 1) recognition, an analogue of today’s credit, and 2) the scientific ethos, a collection of shared norms (communism, universalism, disinterestedness, and organised scepticism, or CUDOS). In the Mertonian paradigm, these two aspects went hand in hand: recognition explained *why* scientists acted, the ethos explained *how* they acted (Storer, 1973, xxiii). In Merton, we can thus see an early predecessor of entanglement: norms and incentives co-constituted behaviour.

Today’s credit theorists often point to Merton’s notion of recognition, but they rarely refer to Merton’s notion of a scientific ethos.^14^ This already makes it questionable that the notion of credit could map one-to-one onto Merton’s account of recognition: if this was the case, we should expect more explicit discussion of the links between the CUDOS norms and the credit economy.

A possible reason for the CEA’s limited attention for Merton’s scientific ethos might be the scrutiny Merton’s notion came under in sociology of science itself. A major criticism was that scientists profess the CUDOS norms when asked, but the observance of these norms could not be detected in the actual behaviours of scientists (Barnes & Dolby, 1970). This critique is another reason why we cannot simply import Merton’s account as the foundation of the CEA.

Consequent sociological analyses tended to focus more on scientific production as a consequence of social processes like struggles for power or negotiation. Notable examples are Bourdieu’s (1975) analysis of science as a struggle for prestige, where the rules for dissemination of prestige are set by the winner of the struggle; Latour and Woolgar’s (1987) account of credit as credibility, which is a capital-like accumulation of ability to participate in the cycles of scientific production; and Bloor’s (1991) account of the norms of rationality as the result of social-causal processes.

There are a number of reasons why these approaches fall short as an underpinning of the CEA. Firstly, although these approaches vary widely in contents, they share an underlying idea that what counts as valuable in a scientific context is the result of a social process like a struggle for power or a negotiation. Especially for Bourdieu and Bloor, norms that pick out certain products as scientifically valuable – true, rational, prestigious – are only formulated post-hoc, after the power struggle has taken place. As a consequence, under these approaches it is hard to *predict* – before the struggle has taken place – what kind of behaviours will be rewarded. But this contradicts a central aspect of the CEA: the credit economy functions exactly because scientists *are* able to predict which behaviours will be rewarded. Thus while Bourdieu and Bloor offer rich descriptions of how prestige circulates in scientific communities, their focus on post-hoc legitimation leaves too little room for the predictive regularities required by a credit economy.

Secondly, being approaches of descriptive sociology, each of these accounts brackets the epistemic aspects of scientific production: the rationalist idea that in the analysis of science we can talk of some unproblematic notion of truth. Indeed, this antirationalist tendency of social analyses of science is one of the reasons why Kitcher first constructed his modelling approach to scientific communities, which lies at the basis of the modern CEA (Kitcher, 1993, e.g. 193–206). On this front too, then, it is hard to wed the CEA to these approaches.

Thirdly, the CEA is committed to methodological individualism: it attempts to analyse social phenomena in terms of the behaviours of individuals. This is shown, for example, in its usage of agent-based models. But methodological individualism clashes with the accounts of Bourdieu and Latour & Woolgar, who offer non-reductive analyses of the social domain in terms of *fields* or *networks*. This methodological difference makes these approaches incompatible with the explanatory ambitions of the CEA.

Most importantly, however, the CEA is much more restrictive in its analyses than any of these sociological accounts. It focuses on a single phenomenon – the priority rule – and asks how the incentive structure that arises from that phenomenon influences the behaviour of scientists. Aspects of sociological analyses can help us make sense of the austere picture that the CEA sketches – like the notion of recognition and the priority rule from Merton, and the notion of cycles of credit from Latour and Woolgar, which can be recognised in the notion of a cycle of esteem and praise that I described in subsect. 3.5. But what was lacking was a coherent picture of how these particular aspects fit together in order to support the assumptions of the CEA. This is what the present paper offers.

In short: although the sociological analyses of science do offer interesting precursors of the notion of entanglement, and make us aware of the inherently social nature of the prestige-related processes of science, they could not be easily imported as the theoretical foundation of the CEA. The account I have sketched is grounded in various aspects of these sociological accounts, but tailored to the CEA as it exists and functions today.

## Conclusion

This paper has studied the relationship between credit-incentives and normative aspects of scientific motivation. I argued that an incentives-only interpretation of the CEA, which casts scientists as merely motivated by career-furtherance, is inadequate. A cumulative interpretation, according to which the effects of norms and values must be added to the effects of credit incentives, is a step in the right direction, but it underestimates the intimate relationship between credit economy and normative motivations. Only by recognising the credit economy’s entanglement with normative motivations can we develop a more accurate and explanatory model of scientific behaviour.

More precisely, I have argued that the credit economy is deeply enmeshed in, and indeed constituted by, the social context, which consists of a complex structure of institutions, shared values, and the psychological makeup of scientists. To capture these insights, I have sketched an account of the prestige-related processes of science based on the notions of esteem and praise which can serve as a theoretical foundation for the CEA. On this account, a central role in the functioning of a scientific community’s credit economy is played by a substantive normative consensus about what it means to be a good member of the community, that is, a good scientist.

The resulting account can help us study questions about the social processes of science which are left untouched by the CEA as it stands today. Is pluralism caused by the norm that a good scientist makes important contributions to their field, or the career-incentives which arise from that norm? Should policy recommendations to “give credit” (Heesen, 2017, 712) for certain behaviours in order to incentivise them be interpreted as an injunction to change our praise-institutions, or to change the social identities which lie at the basis of the esteem-distribution? The CEA as it stands today lacks the conceptual toolkit to ask these questions, but the esteem-praise account can help us answer them.

The discussion of section four has given us a taste of how the account can further our understanding of the social processes of science. I showed that the account offers a place both for an incentive-based and a norm-based understanding of scientific behaviours, and helps us scrutinise their interrelations. We saw how the account sheds light on the normative structure of science, specifically the values that make science tick. And we saw that the psychological notion of social identities can help us understand the notion of the good scientist.

This has far from exhausted the account’s potentials: many future directions of research remain. I have identified discovery as a driving value for the credit economy, but which other values might there be? I have only described an idealised conception of the credit economy; what happens if we introduce imperfections? An especially promising line of inquiry are the consequences of the esteem-praise account for our understanding of science policy. Since the account casts the credit economy as deeply value-laden, it will be interesting to explore how policymakers’ attempts to incentivise certain behaviours by means of interventions in the credit economy affect and interact with the value aspect of the prestige-related processes of science.

The distinction between the normative consensus and the credit institutions clarifies the basis on which those institutions can be criticised. Pathologies of the credit economy are well-known, such as publish-or-perish culture and the difficulty publishing replication studies (see also footnote 9). The notion that a normative consensus gives rise to the credit economy suggests a route for internal criticism of credit institutions: the credit economy is internally good to the extent that it lives up to the ideals embodied in the normative consensus that gives rise to it.

Because the account includes the social-psychological notion of social identities, it explicitly makes space for empirical knowledge of human behaviour to inform our understanding of the social processes of science, which means that we can cast our net for future directions of research even wider. Social-psychological self-categorisation theory is, amongst other things, concerned with explaining the psychological mechanisms which drive the formation of social identities. Insights from this discipline could help us understand under which conditions scientific identities might change, and in which direction such change might happen. Similar insights might help us understand why the priority rule has become a central norm of the scientific world – a fact which the CEA has taken as given. The result, we may hope, will be a much richer and more nuanced understanding of the motivational structures with which scientists are faced than the CEA can currently provide.

What started as a quest to understand the interrelations between incentives and values has thus resulted in much more. Simultaneously, the account of the prestige-related processes that I have presented is still bare-bones, and requires fleshing out. Much is left to do. But with effort, and some luck, a great opportunity lies this way: an integrated theory of the social organisation of the sciences, empirically grounded in a broad range of social sciences – sociology of science, economics, social psychology – and computationally reinforced by formal models. All we need are a few good scientists.

## Data Availability

Not applicable.

## Footnotes

1 See e.g. (Heesen, 2017, 701).

2 Or satisficing with regard to particular thresholds (as in (Heesen, 2024)). The distinction between the two has no significant bearing on the arguments I present.

3 The classic reference for the importance of priority is (Merton, 1957) and as far as I can find, the term “priority rule” was introduced in (Strevens, 2003). Of course, empirical sociological work has shown that prestige can be garnered for other things, but within the formal modelling tradition that is the CEA, credit is very closely associated with the priority rule. This is one of the strong divergences between the CEA and its sociological predecessors (see Sect. 5).

4 This strategy is pursued, for example, in (Cressman et al., 2014).

5 I am talking about “pure credit” prizes, which do not involve any other reward than the honour of receiving the prize. If a monetary reward is associated with the prize this might change our expectations about people’s behaviour, but it is debatable whether this behavioural change would be due to a *credit* incentive, rather than a *monetary* incentive.

6 The two other defining features of esteem for Brennan and Pettit are comparativeness, meaning that the esteem allotted to a certain person is dependent on how they do relatively to others, and directiveness, meaning that esteem is allotted in areas where the agent can be assumed to hold some influence over his or her performance. Both clearly hold for credit, as well.

7 Note that this argument does not fly if one takes a deflationary view of prestige according to which the evaluative measure supposedly underlying the allocation of prestige is really a post-hoc rationalisation of pre-existing power-relations – see the discussion of Bourdieu and Bloor in Sect. 5. But as I argue in that section, it is hard to see how that brand of prestige could serve as the basis of the kind of incentives in which the CEA deals. If credit-incentives are mediated by prestige, then it must be somehow possible to *predict* what kind of activities will render one prestigious – a feat which is significantly more complicated if prestige-allocation is rationalised only on a post-hoc basis.

8 This notion of a cycle of value which assumes various forms is reminiscent of analyses of capital in (Marxian) economics. Latour and Woolgar’s analysis of credit takes this analogy seriously and carries it to its logical conclusions: (Latour et al., 1987, 187–233).

9 Under ideal conditions, a scientist participating in the normative consensus should expect to be esteemed for exactly the activities she deems worthy of the good scientist. In reality, there is an abundance of ways in which the esteem-allocation can become garbled compared to the normative consensus, causing the two decision rules to diverge. For example, the praise-institutions might fail to convey information about some of the activities that are deemed valuable under the normative consensus. This might be the case with replication studies, which are notoriously hard to get published (Gorgolewski et al., 2018). As a result, scientists should not expect to be esteemed for doing replication studies, even though they might agree that doing replication studies is esteem-worthy. One of the virtues of the present account is that it offers us a vocabulary to more accurately capture such “misfiring” of a credit economy.

10 My usage of the term “alienation” is inspired by Bratman’s (2022) treatment of alienation in the context of institutions. Bratman’s account provides one way to cash out the notion of institutions and norms in the present text. I prefer to ground my account in the social-psychological notion of social identities; see Sect. 4.4.

11 My account of the credit economy therefore unifies the two approaches to thinking about the social structure of science that Hugh Desmond characterises, the economic approach and the ethical approach. Using Desmond’s terminology, my argument is that the ethical conception of scientific action underlies and facilitates the economic conception of scientific action. See (Desmond 2021, 906).

12 (Boettke & O’Donnell, 2016) argue that exactly such a norm should guide the actions of economic researchers.

13 See Sect. 4.1 and the qualifications made in note 9.

14 An exception is (Heesen, 2017), which aims to show that the Mertonian norm of Communism can be understood in terms of credit incentives.
